# Lactobacillus-Associated Spontaneous Bacterial Peritonitis in a Liver Cirrhosis Patient on Probiotics

**DOI:** 10.7759/cureus.11896

**Published:** 2020-12-04

**Authors:** Lintu Ramachandran, Venkata S Dontaraju, Kushal Patel

**Affiliations:** 1 Internal Medicine, Javon Bea Hospital, Rockford, USA

**Keywords:** lactobacillus, probiotics, sbp, spontaneous bacterial peritonitis, liver cirrhosis, paracentesis

## Abstract

The efficacy of over the counter probiotics has been an area of scientific debate. While the benefits of probiotics are heavily disputed, probiotics are considered generally safe. We present a case of a liver cirrhosis patient, who presented with hepatic encephalopathy. The patient was taking daily probiotics and receiving weekly therapeutic paracentesis. His workup revealed spontaneous bacterial peritonitis (SBP). Despite starting the patient on empiric ceftriaxone and vancomycin, the patient's leukocytosis did not improve. The paracentesis fluid and blood cultures grew *Lactobacillus gasseri*. Antibiotics were switched to piperacillin/tazobactam, after which the patient improved clinically. The case highlights the importance of vigilance in using probiotics, especially in liver cirrhosis patients. Also, patients with Lactobacillus-associated SBP may not improve with empiric antibiotic treatment of cephalosporins.

## Introduction

Lactobacilli are gram-positive bacilli that live in the gastrointestinal (GI) and vaginal tract of humans. The use of Lactobacilli as a probiotic has been an area of ongoing research for several years. Furthermore, recent studies have investigated the potential cardiovascular, antihypertensive, and weight loss benefits of Lactobacilli [1]. Also, Lactobacillus was given to animal models to prevent spontaneous bacterial peritonitis (SBP) with little success [2]. Despite the current studies, caution needs to be exercised using probiotics, especially with liver cirrhotic patients. We present a case of SBP due to *Lactobacillus gasseri* in a liver cirrhosis patient who was taking daily probiotics.

## Case presentation

Our patient is a 62-year-old male with a past medical history significant for liver cirrhosis from chronic viral hepatitis C and alcohol abuse and refractory ascites despite a transjugular intrahepatic portosystemic shunt (TIPS) procedure requiring weekly paracentesis. The patient also had been taking probiotics for a long time, and he presented with confusion secondary to hepatic encephalopathy. He had a distended, non-tender abdomen with a positive fluid wave test on the physical exam. Labs also revealed leukocytosis. He underwent paracentesis, which yielded 5,200 mL of cloudy yellow fluid. The fluid analysis showed white blood cells (WBCs) with neutrophilic predominance. A CT scan of the abdomen did not show any evidence of GI perforation. He got empiric antibiotic coverage with intravenous ceftriaxone after obtaining the ascitic fluid culture. Despite ceftriaxone, his leukocytosis did not improve. The ascites cultures came back positive for gram-positive bacilli, and ceftriaxone was changed to vancomycin and cefepime. The patient's leukocytosis persisted. Blood culture results then came back positive for *Lactobacillus gasseri*. The antibiotics were changed to piperacillin/tazobactam, and the patient's mentation gradually improved throughout his hospital stay. The patient was discharged on amoxicillin/clavulanic acid based on susceptibilities to complete his antibiotic course.

## Discussion

The term probiotic was coined in 1974 and defined as organisms and substances which contribute to intestinal microbial balance [3]. Since that time, research on probiotics has grown exponentially. Studies have linked the use of probiotics with improved GI health and even increased bone mineral density [4]. There has also been discussion of probiotics as a treatment approach for hepatic encephalopathy [5-8]. As research on probiotics increased, the number of commercially available over-the-counter probiotics has also increased. Our patient was a liver cirrhotic who had been ingesting a daily over­-the-counter probiotic and had SBP from Lactobacillus.

While there have been several case reports of Lactobacilli causing peritonitis in peritoneal dialysis patients, studies reporting pathogenic Lactobacilli in the setting of SBP is minimal at best. There has been one other reported case of Lactobacillus peritonitis in a cirrhotic liver patient. Intestinal hypomotility and localized intestinal immunodeficiency due to cirrhosis were hypothesized to increase bacterial growth and increase mucosal edema, thereby increasing permeability leading to SBP. We hypothesize that our patient's daily intake of probiotics may have contributed to increased Lactobacilli in his gut flora and that the increased gut permeability from his underlying cirrhosis then resulted in his infection.

Lactobacillus-associated peritonitis can also be suspected in patients having leakage of normal flora into the peritoneum after an intra-abdominal perforation. However, in such cases, the peritoneal fluid cultures would be polymicrobial. Moreover, the CT abdomen was negative for any perforation. Another suspected etiology would be Lactobacillus from the skin flora that superseded into the peritoneum from the patient's repeated paracentesis. However, this would also yield polymicrobial results. Our patient's body fluid culture only grew *Lactobacillus gasseri*. 

The antibiotic response to Lactobacilli is highly variable between the different species [9-10]. Due to the limited number of cases of serious infections secondary to Lactobacillus, studies on antibiotic susceptibilities are limited at best. The most common regimens used to treat Lactobacilli are high dose penicillin or ampicillin with or without aminoglycosides. Due to Lactobacillus inherent resistance to vancomycin and cephalosporins, our patient did not improve initially. Our patient responded to piperacillin/tazobactam and improved significantly after appropriate antibiotics. This case demonstrates the importance of early recognition and suspicion of Lactobacillus in patients who do not respond to initial empiric antibiotic therapy for SBP. This becomes furthermore important since Lactobacillus can be gram variable, and they can be intrinsically resistant to most cephalosporins and vancomycin [11].

## Conclusions

In conclusion, our case report illustrates the importance of caution in using probiotics in liver cirrhosis patients. Liver cirrhosis patients ingesting probiotics may be at increased risk of peritonitis from Lactobacilli due to possible translocation. Further studies are necessary to establish the safety and efficacy of probiotics in liver cirrhosis patients. Clinicians should also have a high level of suspicion of Lactobacillus-associated SBP in patients who do not improve with empiric antibiotic treatment. Additional research also needs to be conducted in establishing an empiric antibiotic therapy regimen for Lactobacilli.

## References

[1] Apostolidis E, Kwon YI, Ghaedian R, Shetty K (2007). Fermentation of milk and soymilk by Lactobacillus bulgaricus and Lactobacillus acidophilus enhances functionality for potential dietary management of hyperglycemia and hypertension. Food Biotechnol.

[2] Bauer TM, Fernández J, Navasa M, Vila J, Rodés J (2002). Failure of Lactobacillus spp. to prevent bacterial translocation in a rat model of experimental cirrhosis. J Hepatol.

[3] Fuller R (1992). History and development of probiotics. Probiotics.

[4] Parvaneh K, Jamaluddin R, Karimi G, Erfani R (2014). Effect of probiotics supplementation on bone mineral content and bone mass density. Sci World J.

[5] Bajaj JS, Saeian K, Christensen KM (2008). Probiotic yogurt for the treatment of minimal hepatic encephalopathy. Am J Gastroenterol.

[6] McGee RG, Bakens A, Wiley K, Riordan SM, Webster AC (2011). Probiotics for patients with hepatic encephalopathy. Cochrane Database Syst Rev.

[7] Solga SF (2003). Probiotics can treat hepatic encephalopathy. Med Hypotheses.

[8] Agrawal A, Sharma BC, Sharma P, Sarin SK (2012). Secondary prophylaxis of hepatic encephalopathy in cirrhosis: an open-label, randomized controlled trial of lactulose, probiotics, and no therapy. Am J Gastroenterol.

[9] Delgado S, Flórez AB, Mayo B (2005). Antibiotic susceptibility of Lactobacillus and Bifidobacterium species from the human gastrointestinal tract. Curr Microbiol.

[10] Salminen MK, Rautelin H, Tynkkynen S, Poussa T, Saxelin M, Valtonen V, Järvinen A (2006). Lactobacillus bacteremia, species identification, and antimicrobial susceptibility of 85 blood isolates. Clin Infect Dis.

[11] Gong Y, Li T, Li S (2016). Achieving high yield of lactic acid for antimicrobial characterization in cephalosporin-resistant Lactobacillus by the co-expression of the phosphofructokinase and glucokinase. J Microbiol Biotechnol.

